# Effect of Aging Treatment on the Corrosion Resistance Properties of 7N01 Extrusion Aluminum Alloy

**DOI:** 10.3390/ma14133615

**Published:** 2021-06-28

**Authors:** Yitai Li, Weiou Qin, Shuyuan Yu, Jun La, Yaokun Fu, Jidong Li, Wenchao Yang, Yongzhong Zhan

**Affiliations:** 1 School of Resources, Environment and Materials, Guangxi University, Nanning 530004, China; lyt8590@126.com (Y.L.); 18775837632@163.com (W.Q.); ljsxjzjx@163.com (J.L.); ykfu1117@163.com (Y.F.); lijidong@163.com (J.L.); 2 Guangxi Key Laboratory of Processing for Non-Ferrous Metals and Featured Materials, MOE Key Laboratory of New Processing Technology for Non-Ferrous Metals and Materials, Nanning 530004, China; 3 Shenzhen Customs Industrial Products Inspection Technology Center, Shenzhen 518067, China; szciqysy@126.com

**Keywords:** aluminum alloy corrosion, 7N01 alloy corrosion, corrosion rate, aging treatment, non-isothermal aging

## Abstract

The influences of non-isothermal aging (the temperature range is 150–180 °C, and the heating rate is 5 and 20 °C/h alternately), single-peak aging (aging at 120 °C for 24 h, then water quenched was followed at room temperature), and two-stage aging (aging at 105 °C for 8 h first, then increasing aging temperature to 135 °C and keeping for 12 h, followed by water quenching at room temperature) on the corrosion resistance and microstructure of the 7N01 aluminum alloy under 3.5 wt.% NaCl were investigated using electric polarization curve test and exfoliation corrosion. After aging, the hardness of samples was measured by a Vickers micro-hardness tester, and the electrical conductivities were obtained using the eddy current method. The results show that the steady phase η and metastable phase η′ are precipitated in the grain boundary of 7N01 aluminum alloy after non-isothermal aging, and their distribution is discontinuous. The hardness of the alloy can reach 136.9 HV1 and the electrical conductivity can reach 35.8% IACS, which is close to the hardness of single-peak aging and the conductivity of two-stage aging, respectively. Compared with single-peak aging, the corrosion current density of non-isothermal aging is reduced by 15.5%, and that of two-stage aging is reduced by 28.9%.

## 1. Introduction

High-speed rail has gradually become the main mode of transportation in various countries. A high-speed train body is basically an aluminum alloy profile or plate, and in the subway traffic and light rail traffic, traditional steel has been replaced by an aluminum alloy profile [1]. For the high-speed train base, component framework, frame pillow beam, and other key components of the strength requirements are higher [2,3]. Because of its light weight, high strength, and weldability, 7xxx series aluminum profiles have been used as large bearing members of the electric multiple unit body where the most typical aluminum alloy is 7N01 Al-alloy [4,5]. However, as 7xxx series aluminum alloys, 7N01 Al-alloy also exhibits the problem of poor corrosion resistance [6,7,8,9]. At present, many investigators have found quenching conditions to have an important influence on the structure and properties of age-strengthened aluminum alloys [10]. The retrogression and re-ageing (RRA) process can support the mechanical properties and corrosion resistance necessary to achieve the perfect balance [10]. However, the ageing duration of the RRA treatment process is short, resulting in a certain degree of difficulty in the recovery process of large aluminum profiles [11,12,13]. When heating aluminum profiles with a large thickness, uneven heating temperature is likely to occur. In addition, equipment for a rapid heating and quenching process needs to meet higher requirements to prevent the cracking of large aluminum profiles by precisely controlling quenching stress [14,15,16]. Therefore, a new heat treatment process for a 7N01 Al-alloy large component is extremely necessary.

In recent years, based on previous studies concerning the non-isothermal stage in isothermal heat treatment, some investigators proposed new non-isothermal heat treatment process [14,17,18,19,20]. In contrast with ordinary isothermal heat treatment, the non-isothermal heat treatment process is a heat treatment process works in a changing temperature field by a linear uniform heating and cooling process for the aluminum alloy sample aging treatment. Problems regarding the inconsistent temperatures between inner and surface of large size profiles during the rapid changes of the temperature and heat treatment result in an inhomogeneous microstructure and properties, but these can be solved. With the development of the heat treatment process, from the initial peak aging to subsequent two-stage aging and RRA, precipitated phases can be refined by an aging process, microstructure morphology can be more specific, and properties of the alloy can be revealed [21,22,23]. Non-isothermal processes can be regarded as an infinite number of different isothermal aging stages superimposed [24]. Li et al. found that the corrosion resistance of heat-treated samples ranks in the following order: base metal water quenched oil quenched air quenched [14]. Xiao et al. investigated the corrosion resistance properties of 7050 Al-alloy by non-isothermal aging [25]. However, to the authors′ knowledge, no work concerning the corrosion resistance properties of 7N01 aluminum alloy by non-isothermal aging has been reported. The purpose of this investigation was to study on the corrosion resistance properties of 7N01 aluminum alloy by non-isothermal aging.

## 2. Experiment

### 2.1. Materials

The 7N01 extrusion aluminum alloys were used as the sample material in this investigation. The chemical compositions as listed in Table 1.

After solution heat treatment at 470 ± 3 °C for 60 min, water quenching followed at room temperature, and the solution-treated 7N01 extrusion aluminum alloys were obtained. Then, the samples were subject to single-peak aging, two-stage aging, and non-isothermal aging, respectively. The processing steps of single-peak aging and two-stage aging are listed in Table 2, respectively. The schematic and processing steps of non-isothermal aging are shown in Table 3, respectively. We choose single-peak aging (T6) and two-stage aging (T74), commonly used in production to set the comparison heat treatment process. Before selecting the temperature of the non-isothermal aging process, we conducted a lot of test experiments and found that the conductivity and hardness value increased the fastest in the temperature range of 150–185 °C, so this temperature range was selected for research.

### 2.2. Hardness Measurements

After aging, the samples were polished to smooth and ultrasonic cleaned. The hardness of samples was measured by Vickers micro-hardness tester (HVT-1000, Shenzhen Haoxinda Instruments Co., Ltd., Shenzhen China). During the measurement, the load of the hardness tester is 1 kg, and the load is maintained for 15 s. The size of the hardness test specimen is 15 × 15 × 10 mm. To ensure the accuracy of the experiment, the samples were finely ground with 600#, 800#, 1000#, 2000#, 3000# metallographic sandpaper and polished with 1.5 M type polishing paste until there were no obvious scratches under the optical microscope. At least seven points were selected for each sample for hardness testing. Removing the maximum and minimum values of multiple hardness values at different positions of the sample and taking the remaining hardness data of no less than five, the average value is then taken as the final hardness test result.

### 2.3. Electrical Conductivity Measurements

The electrical conductivities were obtained at room temperature by using eddy current method (Sigma2008 device of the Xiamen Tianyan Instrument Co, Ltd., Xiamen, China) based on DIN EN 2004-1 and ASTM E 1004 standards. Suffice it to say that the electrical conductivities of samples were expressed as a relative value of the International Annealed Copper Standard (%IACS). The size of the sample is: 15 × 15 × 10 mm. Before the measurement, the sample was finely ground with 600#, 800#, 1000#, 2000#, 3000# metallographic sandpaper to make the surface smooth. The test frequency is 60 KHz. The surface of the samples were contacted with a probe until the indication became stable. Each sample needed to be measured three times, and the average value is the final conductivity value of the sample.

### 2.4. Potentiodynamic Polarization Tests

The potentiodynamic polarization tests of samples were carried out in 3.5% NaCl solution with electrochemical workstation (CHI660D, Shanghai Chenhua Instrument Co., Ltd., Shanghai, China). The surface area exposed to the test solution was 1 cm^2^. The Pt wire and KCl-saturated calomel electrode (SCE) were used as the counter and reference electrodes, respectively. The open circuit potential of all samples was monitored for 1 h before the potentiodynamic polarization tests. The scanning of the polarization curves was measured from −2000 mV to 0 mV (versus SCE) at a scan rate of 2 mV/s. The exfoliation corrosion (EXCO) was tested by homogeneous immersion based on ASTM G34-01 standard and the degree of exfoliation corrosion of Al-Zn-Mg-Cu alloys assessed [26]. Table 4 and Table 5 show the spalling corrosion rating details and the degree of corrosion of E grade breakdown level, respectively. The microstructure and corrosion morphology of all samples were examined by scanning electron microscopy experiments with energy dispersive spectroscopy (SU-8020/X-MAX80, Hitachi High-Technologies Corporation, Tokyo, Japan).

## 3. Results and Discussion

### 3.1. Hardness and Electrical Conductivity

Table 6 shows the hardness and conductivity of 7N01 extrusion aluminum alloy after different aging heat treatment processes. The electrical conductivity performance is also often positively related to the corrosion resistance of the material. The principle is that the lattice distortion in the crystal will form a scattering effect on the moving electrons, which hinders the directional movement of the electrons, and thus the conductivity decreases. Appropriate aging treatment can form an appropriate amount of disconnected equilibrium η phase inside the alloy. This precipitated phase is in a non-coherent state with the matrix and causes less lattice distortion, which hinders the directional movement of electrons and reduces the conductance. It can be seen that the hardness of the samples by non-isothermal aging and single-peak aging are very similar, almost 140 HV1. For the conductivity, the treatment with two-stage aging could effectively increase the electrical conductivity of samples. Thus, we initially concluded that the non-isothermal aging could attain the mechanical properties of single-peak aging and corrosion resistance of two-stage aging.

### 3.2. Microstructure of the 7N01 Extrusion Aluminum Alloy After Non-Isothermal Aging

The metastable phase η′ is the main strengthening phase of the Al-Zn-Mg-Cu series alloy. The composition is MgZn_2_, the morphology is disc, the diameter is about 10–20 nm, the thickness is about a few nanometers, and it has a hexagonal structure. During aging, it is formed by the phase transformation of the existing Guinier–Preston (G.P.) zones. zones, and it can also be directly precipitated in the matrix with dislocations and small-angle grain boundaries. The dispersed fine metastable phase η′ phase can greatly improve the strength of the alloy. At elevated temperature or longer aging time, the alloy will form an equilibrium phase η. The main component is MgZn_2_, which is non-coherent with the matrix. Compared with the G.P. zones, the η′ phase is larger in size, with a diameter of 50–60 nm and a thickness of approximately 10–20 nm. The η phase exists in the matrix in the form of flakes, rods, or discs. Because the equilibrium phase η is relatively coarse, when the metastable phase is transformed into the equilibrium phase in a large amount, the strength of the alloy is obviously reduced.

**Figure 1:**
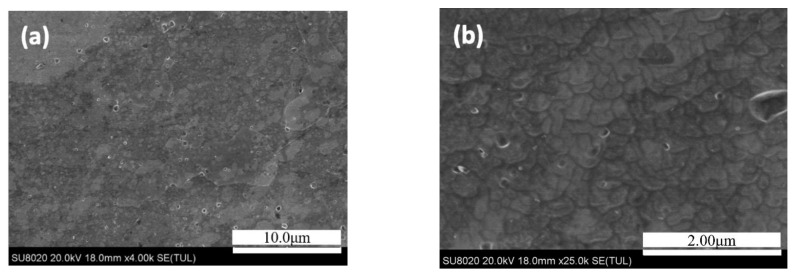
The microstructure morphology and of the 7N01 extrusion aluminum alloy after solution heat treatment: (**a**) SEM images under 4k magnification; (**b**) SEM images under 25k magnification.

**Figure 2:**
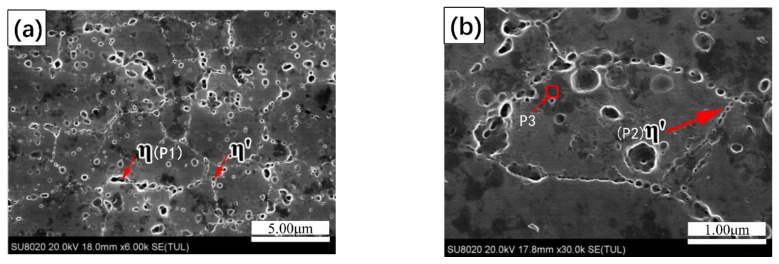

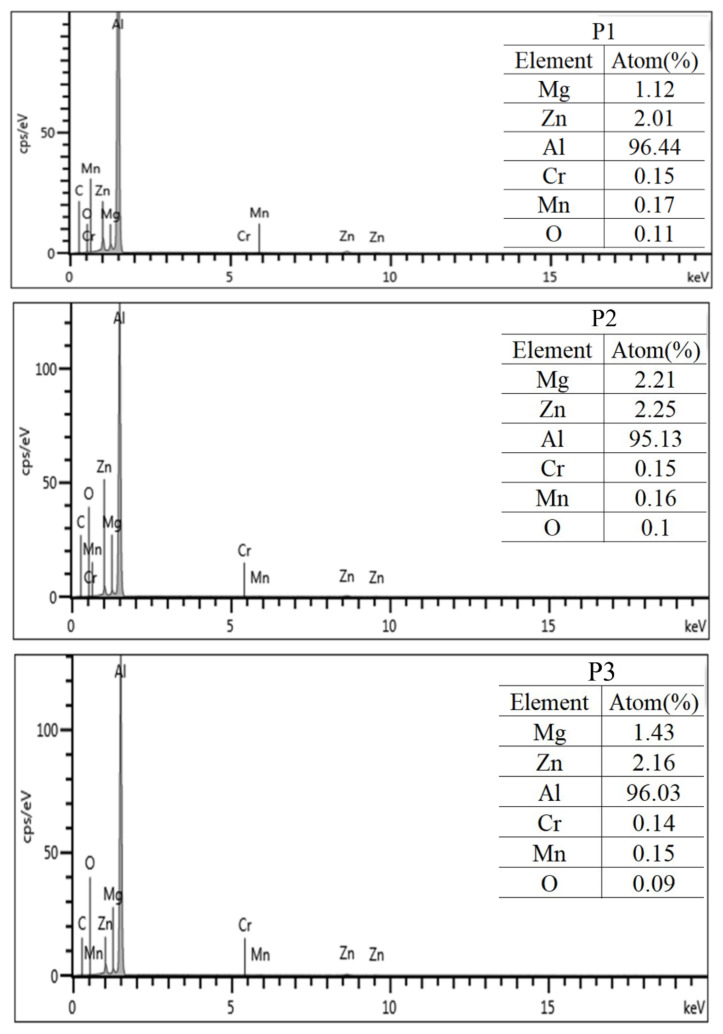
The microstructure morphology and EDS analysis of the 7N01 extrusion aluminum alloy after non-isothermal aging: (**a**) SEM images under 6k magnification; (**b**) SEM images under 30k magnification.

Figure 1 shows the microstructure morphology and the 7N01 extrusion aluminum alloy after solution heat treatment. Figure 2 shows the microstructure morphology and EDS analysis of the 7N01 extrusion aluminum alloy after non-isothermal aging. As shown, the coarse steady-state η phase and relatively small metastable η′ phase precipitate in the grain boundary of the sample at the same time, demonstrating breakaway distribution. The main elements at point P1 are Mg, Zn, and Al, and the rest are impurities with a very small amount. Among them, the atomic number ratio of Mg and Zn is close to 1:2, combined with the larger precipitated phase volume morphology at P1, it can be determined as a steady-state phase. The atomic number ratio of Mg and Zn at P2 is close to 1:1, and it can be inferred that the lighter colored precipitated phase at this point is the coarsened metastable η′ phase. There are three main elements in the P3 region: Mg, Zn, and Al, and the rest are very few impurities. The atomic ratio of Mg and Zn is close to 2:3. Combined with the P3 region in the black region, it can be speculated that the P3 may be the Mg_2_Zn_3_ phase.

### 3.3. Corrosion Resistance

#### 3.3.1. Polarization Curve

**Figure 3:**
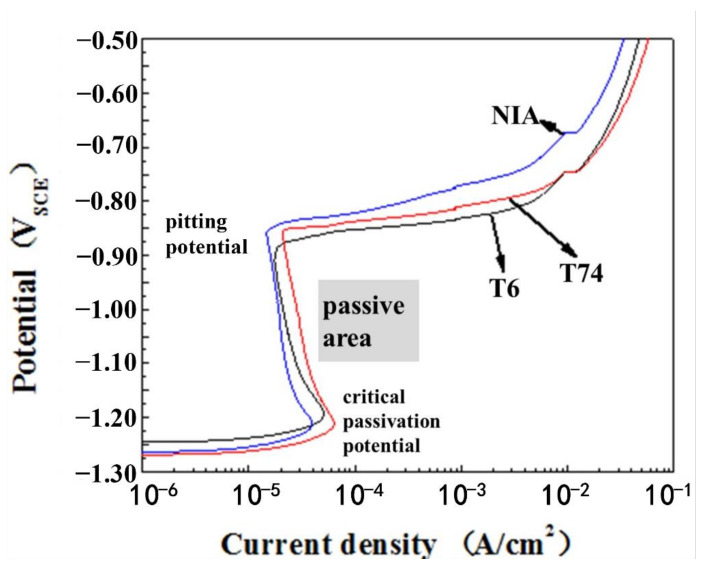
The polarization curves of the7N01 extrusion aluminum alloy after different aging.

Figure 3 shows the dynamic potential of the anode polarization curves of the 7N01 extrusion aluminum alloy after different aging. In the figure, T6 is single-peak aging, T74 is two-stage aging, and NIA is non-isothermal aging. The active dissolution was occurred to the surface of all samples at the beginning of experiment, which presented as the current density increases rapidly with the dissolution of the anode in the stage. When the anode potential rises to the critical passivation potential, the current slowly decreases with increasing of potential and the stage was passivation. The reason for this is that a layer of high resistance value and good corrosion resistance passivation film was produced and stably existed on the surface of the sample. When the corrosion potential was reached, the current density largely increased with a small increase of the potential indicating the breakdown of the passive film. In addition, it should be noted that pitting can be found in the range between −0.75 and −0.65 V.

The correlation data of polarization curves are obtained by Tafel′s extrapolation. The critical passivation potentials of the alloys after the three aging processes are −1.194, −1.211, and −1.212 V, respectively. It can be seen that the difference in value is not very large, indicating that the corrosion resistance of the passivation films produced when they are in a corrosive environment is similar. As shown in Table 7, the corrosion potential of different aging samples were −907 × 10^−3^ V, −859 × 10^−3^ V, and −860 × 10^−3^ V. Generally, the corrosion potential is the positive correlation with the corrosion degree of the sample. The corrosion potential of the sample with single-peak aging was maximum, indicating that its corrosion resistance property was the worst. For the corrosion speed: non-isothermal aging < two-stage aging < single-peak aging. Based on the above analysis, the sample with non-isothermal aging was the best in terms of the level of corrosion resistance.

#### 3.3.2. Exfoliation Corrosion Internal Analysis of Aging

**Figure 4:**
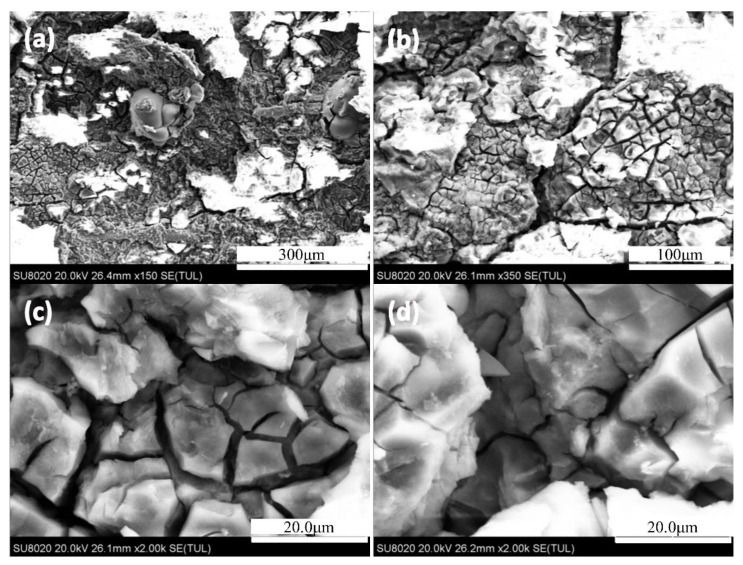
The internal morphology of the 7N01 extrusion aluminum alloy after single-peak aging at infiltrating 24 h: (**a**,**b**) SEM images under 150 magnification; (**c**) cracks in (**b**) under 2k magnification; (**d**) the gully in (**b**) under 2k magnification.

**Figure 5:**
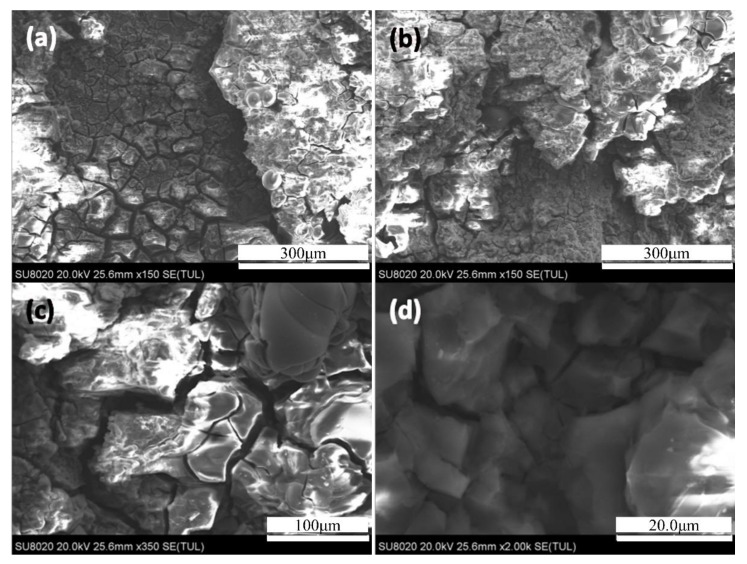
The internal morphology of the 7N01 extrusion aluminum alloy after two-stage aging at infiltrating 24 h: (**a**,**b**) SEM images under 150 magnification; (**c**) SEM images under 350 magnification; (**d**) cracks in (**a**) under 2k magnification.

**Figure 6:**
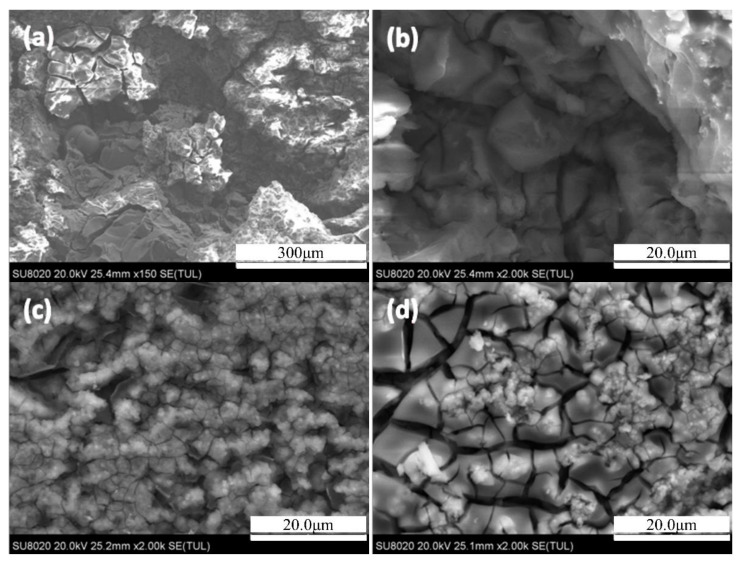
The internal morphology of the 7N01 extrusion aluminum alloy after non-isothermal aging at infiltrating 24 h: (**a**) SEM images under 150 magnification; (**b**) areas with severe corrosion under 2K magnification; (**c**,**d**) the part where corrosion is not obvious under 2K magnification.3.3.3. Corrosion surface morphology.

According to the graph we can be overall exfoliation corrosion assessment, and the comprehensive views of the corrosion morphology were showed in the graphs. For accurate analysis of alloy matrix morphology under the corrosion products, the author cleaned the sample surface of 24 h of infiltration to move out the corrosion products and used scanning electron microscope as substrate surface morphology of in-depth analysis from another aspect of the corrosion principle. Figure 4, Figure 5 and Figure 6 are microscopic, enlarged images of the substrate observed with a scanning electron microscope after the 7N01 extrusion aluminum alloy in different aging states were soaked for 24 h, after removing the corrosion products of the skin and the protrusions of the metal flakes.

Figure 4 shows the internal morphology of the 7N01 extrusion aluminum alloy single-peak aging after 24 h infiltration. It can be seen from Figure 4a,b of low magnification morphology that the corrosion surface morphology of sample with single-peak aging is very serious. The large corrosion pits are a representation of the corroded aluminum matrix that can be seen from the surface to the internal section of matrix (see in Figure 4b–d) are magnification morphology. As was seen in Figure 4c, the metal surface film is divided into a grid of small pieces, which are similar to the microstructure of grain boundaries in Figure 2. Many tiny cracks were found in a ravine of corrosion products in Figure 4d. These gullies are obviously wider than the corrosion cracks produced in Figure 4c. The reason for this is that a large number of connected equilibriums precipitated phase (η phases) are precipitated in some intergranular parts.

Figure 5 shows the internal morphology of the 7N01 extrusion aluminum alloy two-stage aging after infiltrating for 24 h. It can be seen that the corrosion surface of the sample with two-stage aging was lamellar. Generally, the typical feature of exfoliation corrosion had lamellar and bulk corrosion products on the surface. It should be noted that the lamellar cracks also appeared in the grid corrosion below, indicating that the exfoliation corrosion was the main intergranular corrosion. The sample with two-stage aging represents worse corrosion resistance compared with single-peak aging. In addition, the corrosion morphology is lamellar in shape along the grain boundaries from Figure 5c,d.

Internal morphology of the 7N01 extrusion aluminum alloy non-isothermal aging after infiltrating for 24 h is shown in Figure 6. Gridding fine cracks are distributed in both sides of the corrosion crack in Figure 6b. As is seen from Figure 6c,d, the corrosion existed in the sample with non-isothermal aging while the reaction is only slight. It is mainly pitting corrosion, accompanied by a shallow intergranular corrosion network of cracks on the surface of the substrate. In Figure 6c, it can be seen that a certain number of pitting pits appear on the surface of the sample. According to their size, one or several crystal grains on the surface of the sample will fall off the matrix as the surrounding intergranular structure corrodes. In Figure 6d, there are more obvious, reticular, intergranular corrosion cracks. In some parts, there is also a phenomenon of pitting corrosion extending along the intergranular part, causing some of the crystal grains to be squeezed and protruding upward, resulting in bubbles, which belong to the initial stage of exfoliation corrosion.

**Figure 7:**
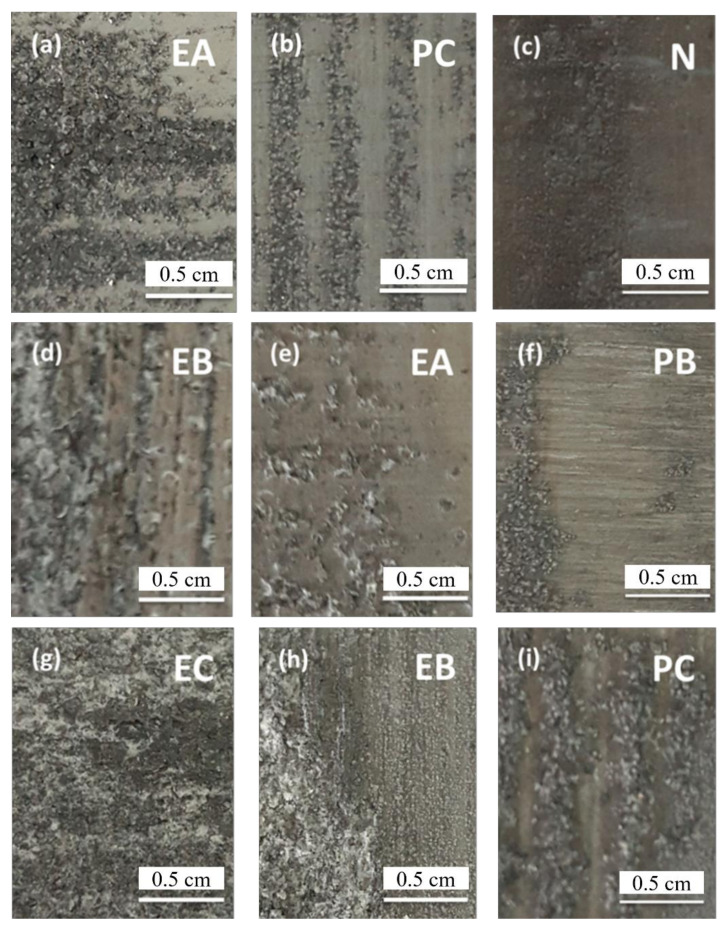
The corrosion morphologies of the 7N01 extrusion aluminum alloy at infiltrated 6 h with different aging: (**a**) single-peak aging; (**b**) two-stage aging; (**c**) non-isothermal aging; the corrosion morphologies of the 7N01 extrusion aluminum alloy at infiltrated 12 h with different aging: (**d**) single-peak aging; (**e**) two-stage aging; (**f**) non-isothermal aging; the corrosion morphologies of the 7N01 aluminum alloy at infiltrated 24 h with different aging: (**g**) single-peak aging; (**h**) two-stage aging; (**i**) non-isothermal aging.

Figure 7 show the corrosion morphology of the 7N01 extrusion aluminum alloy with the different aging. Corrosion rating is shown in Table 8 and Table 9. It can be found that the corrosion morphology of sample with single-peak aging after corrosion tests is most severe, followed by two-stage aging and non-isothermal aging. It is believed that the morphology and distribution of precipitates at the grain boundaries of the alloy are the main reasons for this phenomenon based on the principles of exfoliation corrosion. The sample of single-peak aging at grain boundaries exhibits a continuous chain of η phase [27]. Since these precipitates in the etching process are often preferential corrosion in the alloy grain boundaries and lead to accelerating the progress of corrosion passage, the corrosion resistance property of single-peak aging material is poor. Compared to single-peak aging, two-stage aging corrosion resistance has a certain role to improve, but remains inadequate compared to non-isothermal aging. For this phenomenon, we think that, due to the spalling of etching as a special form of intergranular corrosion, corrosion will develop along the intergranular and show lamellar corrosion and exfoliation.

Raw material was processed by extrusion, resulting in wide and elongated grains. In the etching solution, not only the intergranular corrosion reacted in the surface of the material, but the corrosion product volume was larger than the original volume of metal formed in a considerable force. These resulted in an upward tilt of the metal surface layers. In previous studies [27], equilibrium phases (η phase) were further grown and disconnected from each other due to high temperatures of two-stage aging, but this will produce a worse effect in terms of corrosion resistance. While η phases disconnect from each other to prevent the expansion of corrosion along the grain boundary precipitation phase, precipitates are only etched, so greater damage will be produced. Due to intergranular corrosion generally produced inside the material, the effect on chunks material is little. Therefore, the two-stage aging can improve the corrosion resistance to intergranular corrosion. However, in the process of exfoliation corrosion, this thrust for this thin layer of metal is of extraordinary significance. It will push up the metal surface to form the lamellar exfoliation corrosion, layer by layer, whereby the corrosion pattern continues. So, exfoliation corrosion resistance can be better than single-peak aging but may not have a satisfactory effect. In contrast, various precipitation stages of non-isothermal aging are relatively balanced. Most of the η′ precipitates separate out in the grain boundary, decreasing oversaturation solid solubility. The volume of precipitates was diminished, and the force decreased. Therefore, exfoliation corrosion of non-isothermal aging is difficult and will be greatly reduced.

## 4. Conclusions

The 7N01 extrusion aluminum alloys were subject to different aging process. By studying the hardness, electrical conductivity, and corrosion resistance, the following conclusions can be made:

(1) As the 7N01 extrusion aluminum alloy is subject to non-isothermal aging, the hardness of the alloy can reach 136.9 HV1 and the electrical conductivity can reach 35.8%IACS, which is close to the hardness of single-peak aging and the conductivity of two-stage aging, respectively.
(2) The steady phase η and metastable phase η′ are precipitated in the grain boundary of 7N01 aluminum alloy after non-isothermal aging, and their distribution is discontinuous. The precipitates are mainly composed of fine grains of the metastable phase η′ and a small amount of Mg-rich and Zn-rich atomic layer (GP).
(3) According to the dynamic potential of the anode polarization curves, it can be concluded that the corrosion rate of non-isothermal aging samples is the lowest. Compared with single-peak aging, the corrosion current density of non-isothermal aging is reduced by 15.5%, and that of two-stage aging is reduced by 28.9%. So, the corrosion resistance is better.
(4) In the exfoliation corrosion test, the corrosion degree and the weight loss of the samples treated by the non-isothermal aging process are both lower than those of the two-stage aging and single peak aging. The weight loss of non-isothermal aging compared to the single-peak aging sample was reduced by 93.3%, 59.9%, and 43.1% after aging for six, 12, and 24 h, respectively. Compared with two-stage aging, the weight loss was reduced by 89.1%, 39.2%, and 25.7% after aging for six, 12, and 24 h, respectively. This shows that non-isothermal aging demonstrates the best corrosion performance.

## Figures and Tables

**Table 1:** Chemical compositions of the 7N01 extrusion aluminum alloy (wt.%).

Element	Si	Fe	Cu	Mn	Mg	Cr	Ti	Zr	Zn	Al
7N01	0.055	0.153	0.021	0.33	1.40	0.22	0.088	0.13	4.45	Bal.

**Table 2:** The processing steps of single-peak aging and two-stage aging.

Aging Process	Processing Steps
Single-peak aging	Aging at 120 °C for 24 h, then water quenched was followed at room temperature.
Two-stage aging	Aging at 105 °C for 8 h first, then increasing aging temperature to 135 °C and keeping for 12 h, water quenched was followed finally at room temperature.

**Table 3:** The processing steps of non-isothermal aging.

Stage(h)	Variable Temperature Range (°C)	Heating Rate(°C/h)
0→4.5	60→150	20
4.5→8.5	150→170	5
8.5→9.25	170→185	20
9.25→10	185→170	20
10→14	170→150	5
14→18.5	150→60	20

**Table 4:** The spalling corrosion rating details.

Code	Classification	Descriptions of the Various Classifications
N	No appreciable attack	Surface may be discolored or etched, but no evidence of pitting or exfoliation.
P	Pitting	Discrete pits, sometimes with a tendency for undermining and slight lifting of metal at the pit edges.
EA through ED	Exfoliation	Descriptions are showed in Table 5.

**Table 5:** The degree of corrosion of E grade breakdown level was described.

Code	Descriptions of the Various Classifications
EA	(superficial) tiny blisters, thin slivers, flakes, or powder, with only slight separation of metal.
EB	(moderate) notable layering and penetration into the metal.
EC	(severe) penetration to a considerable depth into the metal.
ED	(very severe) similar to EC except for much greater penetration and loss of metal.

**Table 6:** The hardness and electrical conductivity of 7N01 extrusion aluminum alloy after different aging treatment.

Aging Process	Hardness (HV1)	Electrical Conductivity (%IACS)
Single-peak aging	139.1 ± 1.7	31.4
Two-stage aging	127.8 ± 1.5	37.0
Non-isothermal aging	136.9 ± 2.1	35.8

**Table 7:** The corrosion parameters the 7N01 extrusion aluminum alloy after different aging.

Aging Process	Critical Passivation Potential (V)	Critical Passivation Current Density (A /cm^2^)	Corrosion Potential (V)	Corrosion Current Density (A/cm^2^)
Single-peak aging	−1.194	5.10261 × 10^−5^	−907 × 10^−3^	1.75272 × 10^−5^
Two-stage aging	−1.211	6.37800 × 10^−5^	−859 × 10^−3^	2.08200 × 10^−5^
Non-isothermal aging	−1.212	3.98307 × 10^−5^	−860 × 10^−3^	1.48128 × 10^−5^

**Table 8:** The corrosion rating of the 7N01 extrusion aluminum alloy with different aging after different time period.

Heat Treatment Classification	Exfoliation Corrosion Rating of Different Time Periods		
	6 h	12 h	24 h
Single-peak aging	EA	EB	EC
Two-stage aging	PC	EA	EB
Non-isothermal aging	N	PB	PC

**Table 9:** The corrosion weight loss of the 7N01 extrusion aluminum alloy with different aging after different time periods.

Heat treatment classification	The Weight Loss of The Different Time Periods(g)		
	6 h	12 h	24 h
Single-peak aging	0.1052 ± 0.0023	0.1728 ± 0.0026	0.2455 ± 0.0033
Two-stage aging	0.0651 ± 0.0011	0.1139 ± 0.0014	0.1882 ± 0.0028
Non-isothermal aging	0.0071 ± 0.0016	0.0693 ± 0.0012	0.1398 ± 0.0020

## Data Availability

Data sharing is not applicable to this article.
